# Inhibition of BET bromodomains as a therapeutic strategy for cancer drug discovery

**DOI:** 10.18632/oncotarget.3551

**Published:** 2015-03-12

**Authors:** Lei-lei Fu, Mao Tian, Xiang Li, Jing-jing Li, Jian Huang, Liang Ouyang, Yonghui Zhang, Bo Liu

**Affiliations:** ^1^ State Key Laboratory of Biotherapy, Collaborative Innovation Center of Biotherapy, Department of Urology, West China Hospital, Sichuan University, Chengdu, China; ^2^ School of Traditional Chinese Materia Medica, Shenyang Pharmaceutical University, Shenyang, China; ^3^ Collaborative Innovation Center for Biotherapy, Department of Pharmacology & Pharmaceutical Sciences, School of Medicine, Tsinghua University, Beijing, China

**Keywords:** bromodomain, BRD2/4, BRD3, BRDT, BET inhibitor

## Abstract

As a conserved protein interaction module that recognizes and binds to acetylated lysine, bromodomain (BRD) contains a deep, largely hydrophobic acetyl lysine binding site. Proteins that share the feature of containing two BRDs and an extra-terminal domain belong to BET family, including BRD2, BRD3, BRD4 and BRDT. BET family proteins perform transcription regulatory function under normal conditions, while in cancer, they regulate transcription of several oncogenes, such as c-Myc and Bcl-2. Thus, targeting BET proteins may be a promising strategy, and intense interest of BET proteins has fueled the development of structure-based bromodomain inhibitors in cancer. In this review, we focus on summarizing several small-molecule BET inhibitors and their relevant anti-tumor mechanisms, which would provide a clue for exploiting new targeted BET inhibitors in the future cancer therapy.

## INTRODUCTION

Acetylation of lysine residues is a widespread protein post-translational modification (PTM), and extensively relevant to modulation of cellular processes, including protein conformation and interaction [1]. Histone lysine acetylation was historically proposed to be a hallmark of transcriptionally active genes [2], and hitherto, deregulation of histone acetylation patterns often drives the aberrant expression of oncogenes resulting in proliferation and tumorigenesis [3]. Three types of proteins have been identified to regulate lysine acetylation: bromodomain (BRD) proteins [4, 5], histone acetyltransferases (HATs), histone deacetylases (HDACs) and sirtuins (SIRTs) [6-9]. BRD proteins bind to acetylated lysine (Kac) and thus acting as readers of lysine acetylation state; HATs effect lysine acetylation acting as writers; HDACs and SIRTs remove acetyl groups as erasers [9]. Bromodomains, functioning as acetyl-lysine binding domains, belong to a family of evolutionarily conserved protein modules originally found in proteins associated with chromatin and in nearly all nuclear HATs [10]. BRDs may contribute to highly specific histone acetylation by tethering transcriptional HATs to specific chromosomal sites, or to the activity of multi-protein complexes in chromatin remodeling [11]. Thus, BRDs modulate enzyme activities, protein assembly and protein-protein interactions (PPIs) via lysine acetylation, revealing broad implications for the mechanisms underlying a wide variety of cellular events, such as transcriptional activation and chromatin remodeling [12].

Human genome encodes 61 BRDs in 46 different proteins, in which legend specificity is imparted in the amino acid residue differences around the acetyl-lysine binding site [13]. BRD proteins mostly contain one or two bromodomains, while some proteins, such as nuclear scaffolding proteins (PB1), contain more than two BRDs [14]. Bromodomain and extra-terminal (BET), which taxonomically belongs to human BRD proteins family, shares a common domain architecture comprising two N-terminal bromodomains and an extra-C terminal domain. BET family consists of four mammalian members, including BRD containing 2 (BRD2), BRD3, BRD4 and BRDT, which both exhibit high levels of sequence conservation and a more divergent C-terminal recruitment [14]. Additionally, BET family proteins have been identified in oncogenic rearrangements, leading to highly oncogenic fusion proteins, and thus play key roles in development of several types of cancer. However, it is still unclear why only a subset of cells from diverse types of cancer responds to BET inhibitors [15].

Currently, there have been five registered active clinical trials investigating the targeting of BET family proteins, such as RVX-208, I-BET 762, OTX 015, CPI-0610 and TEN-010, in which OTX 015 has reported encouraging results in treating hematologic malignancies [16-19]. Meanwhile, multiple small-molecule inhibitors of BETs have also been developed and revealed great potential for clinical application, for instance, JQ1 and I-BET both exert the ability to interact with NF-κB and induce apoptosis in drug-resistant leukemia [20]. Hitherto, a number of landmark reports have revealed that the “reader” bromodomains are promising therapeutic targets in cancer. In this review, we summarize a series of small-molecule BET inhibitors and their molecular mechanisms in cancer, which may shed light on exploiting more novel BET inhibitors for future drug discovery.

### STRUCTURE CHARACTERISTICS OF BET PROTEINS

**Figure 1:**
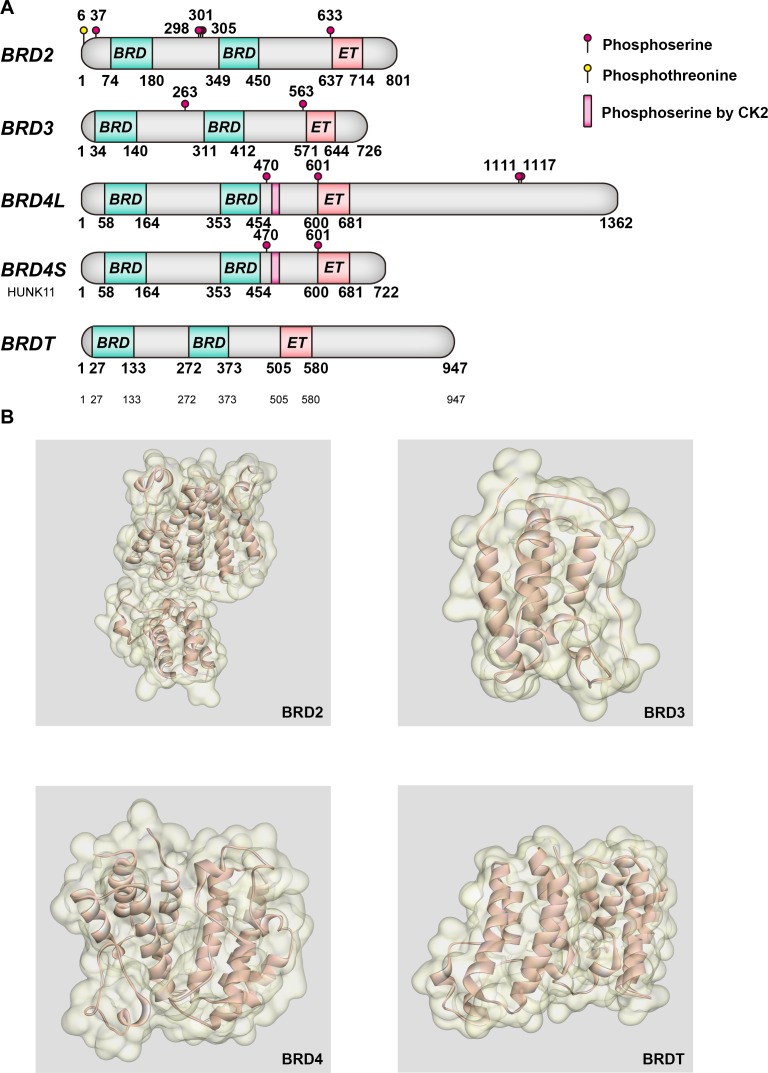
Molecular structures of BET bromodomains Crucial post-translational modification sites and main features of BET proteins. (B) Structure of BET proteins.

BET family proteins all localize in the nucleus, and contain two tandems N-terminal BRDs, an extra-terminal (ET) domain and a more divergent C-terminal recruitment domain, all of which exhibit high levels of sequence conservation. Spanning 61 human BRDs, eight major BRD families are clustered by the derived phylogram analysis, in which BET family belongs to the sub-family II [21]. BRDs' structural analysis of histones lysine-acetylated peptides recognition provides deep insights into characteristics and differences of biological ligand binding selectivity. Not only in BET family, but in all BRDs, it is likely that a hydrogen bond anchor Kac by a conserved asparagine residue their primary role is binding to Kac residues [22]. Besides, the conserved BRD fold contains a deep and largely hydrophobic acetyl lysine binding site, comprised of approximately 110 amino acids. BRDs contain 4 helices αZ, αA, αB, and αC, which form a characteristic antiparallel four-helix bundle, linked by two diverse loop regions, ZA and BC loops [23]. Helix αZ is flanked by a diverse sequence region, whose inserts are typically followed by a short helical segment in the ZA loop. The conserved motif follows the generic sequence Φ_1_X_1_X_2_(X_3_)Φ_2_X_3_X_4_X_5_(X_6_)Φ_3_, as Φ_i_ representing hydrophobic residues, N-terminal domain of BET family members specifically presents insertions at X_3_ [22]. At one end, the N and C termini come together, emphasizing the modular architecture of this domain and underscoring the idea that the BRD could act as a functional unit for PPIs. At the opposite end, the ZA loop packs against the BC loop, forming a central deep hydrophobic cavity that recognized as acetyl-lysine epitopes [24]. High-resolution co-crystal structures have shown that the first acetylated lysine mark of histone H4 docks directly onto the conserved asparagine. Conserved residue phenylalanine of BRDs deeply buried in helix αC stabilizes the C-terminus helical segment, while three conserved proline residues that ZA loop harbors may closely pack to hydrophobic residues in a C stabilizing the loop conformation. Simultaneously, similar as most BRD proteins, X-ray crystallography verifies BD1 of BRD2, 3 and 4 have an isoleucine at the position analogous to residue 162 in BRD2. This ‘gatekeeper’ residue, which varies in size across the BRD family, controls access to a lipophilic region comprising a tryptophan-proline-phenylalanine sequence (WPF shelf) which is present in a number of BRDs [25]. In BRD2, BRD3 and BRD4, affinity between H4 tail and different BRDs varied, for instance, BRD4 (1) seemed to specifically recognize multiple marks found on the H4 tail, while BRD4 (2) interacts with combinations of two and three acetylated lysines. However, studies indicate that BRDT requires two K_ac_ residues for high–affinity binding. The importance of this shelf was also highlighted in BRDT, in which a diacetylated histone H4 tail to BRDT-BD1 and second AcK at position 8 lies across the WPF shelf leading to increased binding affinity [25]. BRDs distinguish different protein binding partners since they hold the sequence diversity in ZA and BC loop regions, binding to residues neighboring Kac in the target protein or peptide [26] (Figure 1).

### BIOLOGICAL FUNCTION OF BET FAMILY

#### BET proteins are protein scaffolds, mitotic bookmarks and cell cycle regulators

**Figure 2:**
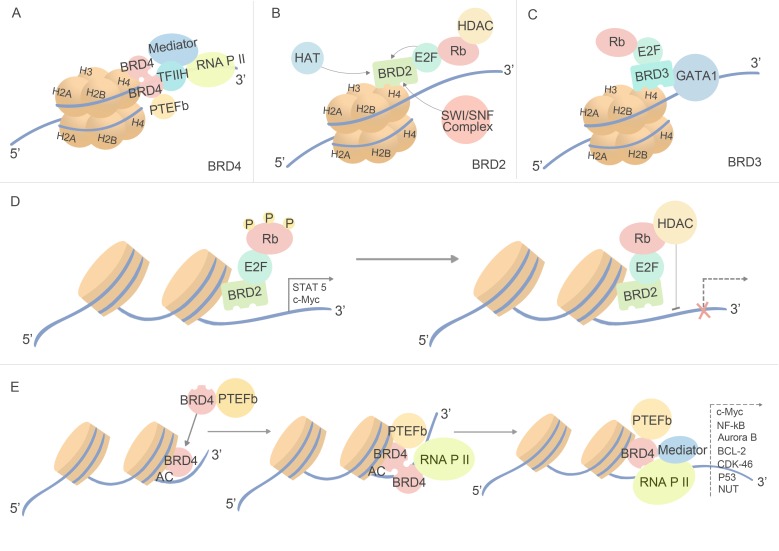
Biological function of BET bromodomains Through sharing the highly similarity of amino acids sequences and all function as protein scaffolds, BRD2, BRD3 and BRD4 recruit different proteins. (A) BRD4 recruits proteins in a PTEFb-dependent manner, and it regulates the transcription process through coupling with RNA P II. (B) (C) BRD2 and BRD3 both exert function through E2F-RB pathway and in a PTEFb-independent manner, while BRD2 distinct from BRD3 for its ability to binding with SWI/SNF complex and regulating binding of ATP and histones. (D) The process how HDAC inhibits transcription through binding with BRD2 and E2F-Rb complex. (E) The process how BRD4 regulate transcription.

Except BRDT specially locating in testis, BET proteins are widely distributed, and exert function to regulate an array of cellular processes. Firstly detected as protein scaffolds, BET family proteins recruit variety proteins to chromatin and transcription sites. During interphase, BRD4 recruits positive transcriptional elongation factor complex (PTEFb) to sites of active transcription, while another pool of BRD4 may be recruited by transcription mediator complexes independent of PTEFb [27]. In addition, the ET domain of BRD4 independently recruits transcription-modifying factors, including glioma tumor suppressor candidate region gene 1 (GLTSCR1); NSD3, a SET domain-containing histone methyltransferase; JMJD6, a histone arginine demethylase; and CHD4, a catalytic component of the NuRD nucleosome remodeling complex [28]. (Figure 2A) Different from BRD4, BRD2 binds to ε-aminoacetyl groups of nucleosomal histone lysines in a PTEFb-independent manner, and with related bromodomain proteins it provides a scaffold on chromatin to recruit E2F proteins, histone deacetylases (HDACs), histone H4-specific acetyltransferase (HAT) and proteins involved in chromatin remodeling, thereby coupling histone acetylation to transcription [29] (Figure 2B). Similar with BRD2, BRD3 may regulate cell processes through E2F-RB pathway, moreover, it can directly recognized acetylated transcription factor, GATA1, which is essential for the targeting of GATA1 to chromatin [30] (Figure 2C).

BET family may also function as mitotic bookmark, identifying actively transcribed genes during mitosis by remaining associated chromatin when all the other factors dissociate. BRD4 remains associated with H4K5ac histones on chromatin during mitosis, leading to rapid de-compaction of the surrounding chromatin and to transcription post-mitotically [31]. BRD4 marks the start sites of many M/G1 genes, and accelerates expression of G1 genes and promotes cell cycle progression to S phase [32] BRD4 seems to be required for the G2 to M phase transition of the cell cycle because microinjection of BRD4-specific antibodies leads to cell cycle arrest [33] Then, BET family proteins function as cell cycle regulators, as mentioned before, that key transcriptional regulators genes of S phase, E2F1 and E2F2, are associated with BRD2 multi-protein complexes [33]. BRD3-dependent functional relationships with the cell cycle control machinery in normal cells are poorly understood, although forced expression of BRD3 down-regulates the RB–E2F pathway in nasopharyngeal carcinoma cells [33].

#### BET proteins function as transcription regulators

BET family has a pivotal role in regulating the transcription of growth-promoting genes [34], for instance, BRD4 and BRD2 are key mediators of transcriptional elongation by recruiting the PTEFb, which is composed of cyclin-dependent kinase 9 (CDK9) and its activator cyclin T. Productive transcription depends on the phosphorylation of the C-terminal repeat domain (CTD) of RNA polymerase II (RNAP II), and phosphorylation of the CTD residues Serine 5 (Ser5) and Serine 2 (Ser2) is necessary for the recruitment of RNA capping and splicing factors [35]. CTD Ser5 residues are phosphorylated primarily by the CDK7 kinase component of TFIIH, while subsequent Ser2 phosphorylation release RNAP II from an early elongation block and are necessary during productive elongation [36]. During elongation, Ser2 is phosphorylated by PTEFb, which depends on BRD4 for its nuclear localization and activation of its CDK9 kinase subunit [36]. BRD4 acts by affecting an acetylation and PTEFb dependent switch from basal transcription of immature unspliced transcripts to high levels of active mature mRNA. BRD4 is part of the transcription pre-initiation complex and remains associated with the RNAP II transcription complex until productive elongation, and it directly phosphorylates Ser2 while PTEFb only phosphorylates Ser2 if Ser5 has not been previously phosphorylated [25, 31]. Conversely, BRD4 is phosphorylated and activated by PTEFb. In addition, the same as TFIIH and PTEFb, BRD4 can directly interact with TAF7, a general transcription factor that regulates all three CTD kinases [36] (Figure 2C). BRD2 regulates transcription through E2F-RB pathway, while HDAC can phosphorylate RB to arrest the transcription process [37]. The involvement of BRD3 in cancer showed its role in the certain NMC translocations, and BRD3 can potentially associated with MLL fusion oncoproteins in leukemogenesis [38]. Transcriptional inhibition is correlated with the prevention of signal-induced BRD-2, -3 and -4 protein recruitment to affected gene promoters. The inhibitor of BET selectively attenuated the induction of secondary response genes characterized by low CpG content, low basal H3 and H4 acetylation levels, low H3K4me3 methylation and low RNA polymerase II occupancy, suggesting BET proteins are involved in the recognition of gene promoters containing a combination of post-translational histone marks characteristic of poised but inactive secondary response genes [39]. BRD4 regulates NF-κB -dependent genes by binding acetylated RelA subunit of NF-kB [40]. The only specially expressed protein, BRDT, named as bromodomain testis-specific protein, has been reported in several stages of spermatogenesis. BRDT directly regulates activation of transcriptional repressors and activators in spermatocytes, and interacts with hyper-acetylated histone H4 tails to induce condensation of acetylated chromatin of haploid spermatids. In addition, it possesses similar functions of BRD4 in the recruitment of PTEFb to form PTEFb complex [38,100]. Rapid development of both biological function and structural basis of BET protein has made it a newly emerging agent for therapeutic strategy of cancer. Development of small-molecule compounds targeting BET bromodomains has won increasingly attentions by biomedical researchers into this field.

#### BET proteins function in cancer

Histone acetylation levels have been associated with an open chromatin architecture and transcriptional activation, but specific marks have been linked to chromatin condensation [41], regulation of metabolism [42] and DNA repair [43]. Therefore, inappropriate acetylation levels have been associated with an aberrant transcription of disease-promoting genes, including cancer-related genes [44]. BET family proteins have been reported to be involved in a variety of malignant tumors, between which NUT-NMC have been reported to closely related to BRD proteins, in which the BRD-NUT blocks cellular differentiation, and depletion of this oncogene in squamous differentiation and cell cycle arrest. NUT midline carcinoma (NMC), an aggressive squamous cell carcinoma, is accordance with acquired chromosomal rearrangements involving NUT, creating chimeric genes that encode fusion proteins. Usually BRD4-NUT fusion genes are been detected, and less commonly NUT-variant fusion genes involving BRD3 also exists, leading to the expression of BRD-NUT fusion proteins [45].

BET family proteins have reported to able to promote aberrant gene expression in leukemia. MYC-family transcription factors are key regulators of cell growth and survival, whose gene amplification is a common copy-number alteration in cancer, while over-expression or translocation of the MYC locus contributes to Myc activity deregulation. In hematologic cancer models, such as MLL-fusion leukemia [46], acute myeloid leukemia (AML) [47], Burkitt's lymphoma [48], multiple myeloma [49], and B-cell acute lymphoblastic (BLL) leukemia [50], amplification of onco-protein Myc drives distinct transcription programs, and leads to a consequence of cell proliferation. BET family directly regulate the expression of MYC genes, and directly silencing MYC gene expression via disruption of BET protein binding at the MYC locus may largely reduce cell proliferation [51]. Amplification or over-expression of MYC is frequently observed in lung cancer, ovarian cancer and breast cancer [52]. By recruiting a histone H3K36 methytransferase, WHSC1, BET protein BRD3/4 plays a key role in tamoxifen resistance to the ESR1 gene. WHSC1 is critical for maintaining estrogen signaling in ER-positive cells via forming a positive feedback regulatory loop with ERα, which is overexpressed in breast cancer. BRD3/4 interacts with WHSC1 in BRD regions, recognizes acetylated lysines on histone tails of the ESR1 promoter and recruits WHSC1, promoting ESR1 transcription [53]. Recent studies have unraveled a possible mechanism about how BRD4 proteins are involved in the transcription of active genes in cancers, especially associated with a subset of these genes. As previous mentioned, BRD4 and Mediator form a complex in transcription process, this complex may related to super-enhancers, which are span large genomic regions and contain exceptional amounts of Mediator and BRD4. In addition, important tumor genes are also associated with super-enhancers, so far has been identified in myeloma, small-cell lung cancer and glioblastoma. Therefore, BRD4 may regulate ongenetic drivers, such as MYC, through occupying super-enhancers, while inhibition of BRD4 also leads to preferential disruption of super-enhancers and selective loss of oncogene expression [70].

Cancers of neural origin may be related to the distinct expression of BET proteins including glioblastoma, medulloblastoma, and neuroblastoma. For instance, neuroblastoma is a pediatric solid tumor associated with a high frequency of MYCN amplifications, and inhibition of BET proteins in neuroblastoma leads to cell arrest [54]. BET proteins are also required for glioblastoma cell proliferation, mRNA of BRD2 and BRD4 are significantly overexpressed in glioblastoma, while disruption of BRD4 expression reduced glioblastoma cell cycle progression [55]. In melanoma, tumor progression may contribute to epigenetic changes, thus epigenetic and/or transcriptional regulation of certain target genes may support melanoma tumor-genesis. NF-κB regulates cytokine and chemokine production in melanoma, and is believed to contribute to progression of the disease by up-regulation of cell cycle and anti-apoptotic genes [56]. BRD2 and BRD4 are overexpressed in human primary and metastatic melanomas, whose inhibition resulted in down-regulating production of cytokines such as IL-6 and IL-8 [57].

The largely hydrophobic nature of the central acetylated lysine binding pocket and special extra-terminal domain of BET are necessary to accommodate the charge-neutralized acetylated lysine and recruits related proteins, making these modules particularly attractive for the development of inhibitors. Many proteins that use BRDs for their recruitment to specific regulatory complexes have been implicated in the development of cancer [58, 45]. The key principle that identification of small-molecule inhibitors with micromolar affinities has been identified, and recently two selective nanomolar inhibitors of BET proteins, JQ1 and I-BET, have been reported [59, 60]. Increased studies have focus on exploring new small-molecule compounds to selectively inhibit BET bromodomains for cancer therapy, and to date, several compounds have been found to exert their remarkable anti-cancer capacities (Table 1).

**Table 1:** BET bromodomain inhibitors in cancer

name	Cancer type	Target	Mechanism/pathway	Reference
JQ1	Tam-R breast cancer	BRD3/4	Suppresses the classic estrogen receptor-α signaling pathway and the growth of Tam-R breast cancer cells in culture	[53]
	NUT midline carcinoma (NMC)	BRD3/4	Suppresses different BRD4-NUT translocations	[73]
	AML cells	BRD4	Reduce binding of BRD4 and RNA polymerase II to the DNA of c-MYC and BCL2	[62]
	OCI-AML3 cell line	BRD4	Trigger caspase 3/7-mediated apoptosis and DNA damage response.	[63]
	Erythroleukemia cell line UT7	BRD4	Inhibit Epo-induced UT7 proliferation and restoring terminal erythroid differentiation	[95]
	B-cell non-Hodgkin lymphoma	BRD4	Induce cell death through MYC-CYCLON pathway	[96]
	Neuroblastoma	BRD4	Induce cell death through targeting MYCN	[67]
	Primary glioblastoma xenograft lines	BRD4	Induced marked G1 cell-cycle arrest and apoptosis through Bcl-xL and p21(CIP1/WAF1).	[97]
	Osteosarcoma cells	BRD4	Trigger transcriptional silencing of MYC and RUNX2, resulting from the depletion of BRD4	[98]
		BRD2	Decrease STAT5-dependent transcription of both heterologous reporter genes and endogenous STAT5 target genes	[64]
I-BET151	Myeloma cell	BRD2/3/4	Induce apoptosis and exerts strong anti-proliferative effect associating with contrasting effects on oncogenic MYC and HEXIM1, and inhibit transcriptional activator PTEFb	[77]
	AML	BRD4	Suppress cell growth in a HOX gene independent manner, but relieving upon NPM1c mutation and cytosplasmic dislocation.	[79]
	Erythroleukemic (HEL) cell	BRD4	Suppress myeloproliferative neoplasms by constitutively active JAK2 kinase	[78]
I-BET762	Myeloma cell	BRD2/3/4	Inhibit myeloma cell proliferation, resulting in survival advantage in a systemic myeloma xenograft model.	[77]
	neuroblastoma tumor models	BRD2/3/4	Suppress cell growth in apoptosis signaling, and N-Myc-driven pathways, including the direct suppression of BCL2 and MYCN.	[76]
CPI203	Mantle cell lymphoma (MCL)	BRD2/3/4;	Decreased tumor burden, involving simultaneous MYC and IRF4 downregulation and apoptosis induction.	[81]
RVX2135	Myc-induced murine lymphoma	BRD2/3/4	Exhibit broad transcriptional effects in Myc-transgenic lymphoma cells affecting many transcription factor networks.	[82]
Dinaciclib	leukemia	BRDT,CDKs;	Interact with the acetyl-lysine recognition site of the bromodomain testis-specific protein BRDT	[94]
PFI-1	Leukemia	BRD2/4	Induce G1 cell-cycle arrest, downregulation of MYC expression, downregulation of Aurora B kinase	[84]
RVX-208	;	BRD3(BD2);	Raise apoA-I and increase preβ-HDL particles. Displace BET proteins from chromatin modestly affecting BET-dependent gene transcription.	[90]

### SMALL-MOLECULE INHIBITORS OF BET BROMODOMAINS IN CANCER

#### BRD2/4 Inhibitors

**Figure 3:**
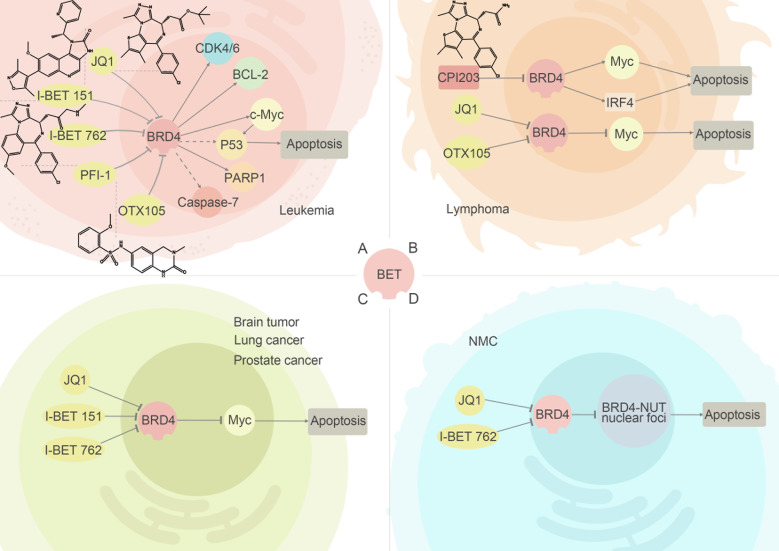
BRD2/4 inhibitors and their relevant anti-cancer pathways Inhibitors of BRD2 and BRD4 have been detected to exert the anti-cancer ability in leukemia, lymphoma, NMC and other tumors. (A) In leukemia, inhibiting BRD4 to regulate transcription of c-Myc can effectively suppress the cell growth. (B) (C) In lymphoma and other solid tumors, inhibiting of BRD2/4 can lead to low expression of specific oncogenes, making BET inhibitors attractive anti-cancer agents. (D) In NMC, suppression of BRD4 can inhibit emerging of BRD4-NUT fusion proteins.

The close similarity, about 80% at the amino acid level in human, between the bromodomains of BRD2 and BRD4 implies that two proteins may share a highly substantial functional similarity. Identified functional studies have verified that both BRD2 and BRD4 play crucial roles in transcription regulation, chromatin remodeling and recruiting related-protein, although the specific proteins may vary. (Figure 3).

Due to the special structure interface of BRD4 protein, targeting bromodomain of BRD4 to inhibit the activity of BRD4-NUT fused protein become an attractive therapeutic strategy. With extensive researches, a small molecular compound, JQ1, exerts both strong inhibition efficiency and highly absorption rate towards NMC. JQ1 is a novel thieno-triazolo-1, 4-diazepine, whose competitive binding to acetyl-lysine recognition motifs enables it a successful anti-proliferative agent in BRD4 dependent cell lines. The bulky t-butyl ester functional group at C6 also allows it additional pendant group diversity and less binding to the central benazodiaepine receptor [59]. Additionally, results of screen of diverse inhibitors of BRD4-NUT and laboratory experiments confirmed JQ1 may process a better efficiency, comparing to the counterpart of other clinic drugs, suggesting its potential in future preclinical studies [61].

Since the crucial role BET proteins plays in leukemia, JQ1 also possesses significant anti-proliferative effect in leukemia, such as AML, T-cell ALL. In addition, JQ1 can inhibit growth and induce apoptosis of human AML cells, including those expressing FLT3-ITD(FMS-like tyrosine kinase 3-internal tandem duplication),a mutation of proto-oncogene. Recent studies also shows that co-treatment of JQ1 and FLT3-inhibitor, FLT3-TKI, can greatly attenuate the expression of c-MYC, BCL2, and CDK4/6, meanwhile synergistically induce apoptosis of cultured and primary CD34(+) human AML blast progenitor cells (BPC) expressing [62]. Activation of caspase 3/7, but not caspase 8, is found in JQ1-mediated apoptosis, indicating that the intrinsic apoptotic pathway is involved [63].

In other hematologic malignancies, JQ1 may exert anti-tumor ability through targeting BRD2, as BRD2 is the critical mediator for STAT5 activity. STAT5, a transcription factor signal, is constitutively activated and drives the expression of genes necessary for proliferation, survival, and self-renewal in leukemia. In experimental models of acute T-cell lymphoblastic leukemia, JQ1 decreases STAT5-dependent (but not STAT3-dependent) transcription of both heterologous reporter genes and endogenous STAT5 target genes, and shows strong synergy with tyrosine kinase inhibitors (TKI) in inducing apoptosis in leukemia cells [64].

BET family proteins may participate in the malignant brain tumor gene regulation, for instance, high-level BRD4 expression was detected in 99 of 115 pediatric primary medulloblastomas (75%) while it was only marginally (22%) expressed in normal cerebellar tissue. One significant clinical challenge for medulloblastoma in pediatric oncology stands that the overall survival currently remains under 70%, and patients with tumors overexpressing MYC or harboring MYC oncogene amplification have an extremely poor prognosis [65]. JQ1 treatment can significantly reduce cell proliferation and induce apoptosis and senescence in different human medulloblastoma cells. Those MYC-amplified cell lines, HD-MB3, ONS-76 and D-341, produced the strongest apoptotic response, while the expression of MYC-associated proteins, such as Cyclin D1 and E2F1, were reduced as well, suggesting JQ1 may exert its anti-tumor ability by targeting BRD4-Myc pathway [66]. Similarly in human neuroblastoma, JQ1 targeted BRD4 to regulate MYCN expression, and induced cell death [67]. In addition, JQ1 inhibited expression of BRD3 and BRD4, and suppressed the classic estrogen receptor-α signaling pathway, resulting in the growth suppression of Tamoxifen resistant breast cancer cells in culture [53].

Particularly in virus-induced tumors, inhibition of host-encoded BET regulates both transcriptional activation and transcriptional repression of virus promoters, making JQ1 a promising therapeutic agent as well. In etiology study of human T cell leukemia virus 1 (HTLV-1)-mediated adult T cell leukemia, JQ1 suppressed the proliferation of Tax-expressing rat fibroblasts, and inhibited transformation and tumor-genesis of Tax-positive HTLV-1-infected cells and Tax-mediated cell. Considering Tax can induce the acetylation of lysine 310 of RelA and promote BRD4 binding acetylated RelA, inhibition of BRD4 by JQ1 may suppress Tax-mediated transcriptional activation of NF-κB [68]. JQ1 treatment can reduce Epstein–Barr virus (EBV) promoter in lymphomas. EBV is the causative agent of lympho-proliferative diseases, whose nuclear antigen (EBNA) proteins providing a functional analogue of LANA1 (Latency-associated nuclear antigen). During the transcription elongation of EBV, inhibition of host-encoded BRD4 can suppress recruiting of PTEFb to viral C promoter, therefore negatively controlling the replication of EBV [69]. JQ1 treatment reduces BRD4 association with the promoter, providing a promising future of drug development in the treatment of latent virus infections by disrupting transcriptional co-regulator.

BET inhibitor, OTX 015, shows the clinically meaningful activity at nontoxic doses, in which inter results of an ongoing phase I trial in hematologic malignances are inspiring. Besides regulating the BRD4, OTX 015 can inhibit the growth of hematologic malignances through directly regulating MYC expression and activity [16]. Besides that, currently, OTX015 has been reported to exert anti-proliferative activity in diffuse large B-cell lymphoma (DLBCL). OTX 015 is highly sensitive to majority (9 of 13) of DLBCL cell lines, and suppression of MYC caused by OTX015 is reversible in DLBCL [71].

GSK525762A (I-BET762) is a specific and potent inhibitor of BET protein binding to acetylated bromodomains, and GSK has embarked it on Phase I clinical trial of NMC. It is derived from medicinal chemistry optimization of a hit derived from a phenotypic screen to identify small molecules able to enhance ApoA1 expression [72] The fusion between BRD4 (and to a lesser extent BRD3) with NUT gene leads to NMC [73], and this fusion is oncogenic due to the inability to sequester important regulatory molecules such as CBP/p300 into BRD4-NUT nuclear foci, which are formed in a bromodomain-dependent manner, and knockdown of BRD4-NUT with anti-NUT siRNAs has been shown to lead to cell differentiation and apoptosis [74]. Treatment of patient-derived samples with I-BET762 has been shown to lead to terminal differentiation and growth arrest of the malignant cells [74]. In addition, I-BET762 potently reduced MYC expression in LNCaP prostate cancer cell lines and a patient-derived tumor model with subsequent inhibition of cell growth and reduction of tumor burden in vivo [75]. In neuroblastoma tumor models, I-BET 762 can trigger apoptosis through BET inhibition of N-Myc-driven pathways, including the direct suppression of Bcl-2 and MYCN. And conversely, reversal of MYCN or BCL2 suppression reduces the potency of I-BET726-induced cytotoxicity in a cell line-specific manner [76]. Further, I-BET762 can influence myeloma cell proliferation, resulting in survival advantage in a systemic myeloma xenograft model [77].

Similar with JQ1, non-benzodiazepine I-BET151 is a pan-BET inhibitor, which can exhibit significant anti-tumor activity in murine models of NUT midline carcinoma, multiple myeloma, MLL and ALL, lung cancer, and malignant brain tumor. In cellular studies of myelo-proliferative neoplasms driven by mutant JAK2 (JAK2V617F), I-BET151 possessed growth inhibitory activity with concomitant down-regulation of LMO2, an important oncogenic regulator of hematopoietic stem cell development [78]. In acute myeloid leukemia (AML) with mutations of the nucleophosmin gene (NPM1), I-BET 151 treatment may down-regulate the core transcriptional program, which is HOX gene independent underlies sensitivity to I-BET treatment [79]. Similar with I-BET762, I-BET151 also inhibits myeloma cell proliferation through inducing apoptosis and exerting strong anti-proliferative effect *in vitro* and *in vivo* through transcriptional repression of MYC and MYC-dependent programs by abrogating recruitment to transcriptional activator PTEFb [77]. BRD2 is the main BET protein involved in regulation of NF-kB and that I-BET151 caused transcriptional downregulation of the NF-kB subunit p105/p50 [80].

CPI203, a BET bromodomain inhibitor, can affect the lymphoma cell growth. The development of Bortezomib resistance to proteasome inhibition in mantle cell lymphoma (MCL) may limit its efficacy of clinical activity. An increased tumorigenicity of bortezomib-resistant MCL cells, which is associated with plasmacytic differentiation features, like interferon regulatory factor 4 (IRF4) and Blimp-1 up-regulation. Repression of the IRF4 target gene MYC in bortezomib-resistant cells by gene knockdown or treatment with CPI203 synergistically induced cell death when combined with lenalidomide [81]. In mice, addition of CPI203 to lenalidomide therapy further decreased tumor burden, involving simultaneous MYC and IRF4 down-regulation and apoptosis induction [81]. RVX2135, a novel and orally bioavailable selective pan-BET inhibitor, presented anti-proliferative ability in Myc-induced lymphoma. What's more, RVX2135 was reported that broad transcriptional changes are mediated, while these are genetically and functionally linked to histone deacetylase inhibitors [82].

PFI-1, a novel dihydroquinazolinone reported as a BET chemical probe, binds to BET bromodomain chemically distinct from previously reported BET inhibitors. Exposure of leukemia cells to PFI-1 results in induction of caspase-dependent apoptosis, differentiation and in down-regulation of the Aurora B kinase. Aurora kinases are highly expressed in diverse cancer types and are also frequently up-regulated in leukemia [83]. In the BET inhibitor sensitive cell line MV4, researchers observed strong induction of PARP1 and pro-caspase 7 cleavage after 24 h exposure with PFI-1 [84]. PFI-1 and JQ1 dissociate BRD4 from HOXA9 and promotes differentiation, as a marker of poor prognosis in patients with acute myeloid leukemia [85] and overexpression of HOXA9 leads to expansion of hematopoietic stem cells in bone marrow cells and development of leukemia in mice [84, 85].

Further, more efficient dual kinase-bromodomain inhibitors have been developed for rationally designed polypharmacology. For instance, two nanomolar activities on BRD4 inhibitors, BI-2536 and TG-101348, have been identified to inhibit bromodomains with therapeutically relevant potencies, particularly noteworthy as shedding light on independent oncogenic pathways [99].

#### BRD3 Inhibitors

Diverse from BRD2-dependent roles in regulating differentiation of adipose tissue and neurons, BRD3 mainly functions in recruitment of GATA1 in hematopoietic cells through regulating maturation of erythroid, megakaryocyte, and mast cell lineages [86, 87]. Inhibitors of BRD3 are less studied than their counterparts in BRD2 and BRD4, due to the lacking of specific mechanism of BRD3. However, pan-BET inhibitors, like JQ1 and I-BET-151, have been found to target BRD3 in NMC and leukemia [88], and inhibition with an I-BET762 analogue led to disruption of normal erythroid maturation.

**Figure 4:**
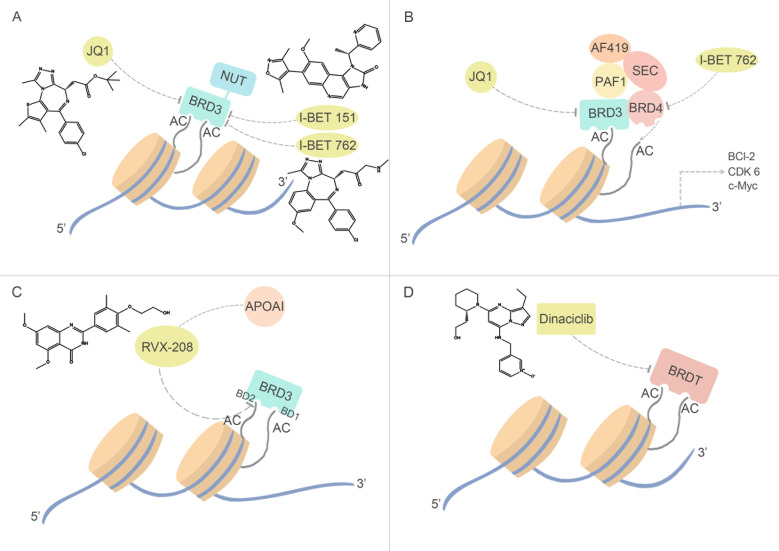
BRD3 and BRDT inhibitors and their relevant anti-cancer pathways (A) Fused with NUT gene, BRD3 may encode BRD3-NUT fusion proteins in NMC, which is similar with BRD4. Specifically inhibiting BRD3-NUT fusion protein can block BRD3-NUT gene transcription, and inhibit NMC cell growth. (B) Pan-inhibitors I-BET762 and JQ1 inhibits BRD3. (C) As the only member of BET family can bind with transcription factor GATA1, inhibition of BRD3 through BRD3-GATA pathway by clinical experimental compound, RVX-208, may be a therapeutic strategy in hepatocellular carcinoma. (D) The least known BET family member, BRDT, is also been detected the anti-cancer capacity in leukemia by an unspecific-target drug Dinaciclib.

Currently, a disappointing result of negative clinical finding of RVX-208 has been reported, which is acting as an ApoA1 modulator in phase I/II clinical trials for the treatment of cardiovascular diseases [89] The quinazolone RVX-208, a derivative of the plant polyphenol resveratrol, acts as interaction partner of ApoA1 and performs a preferentially binding ability to the BD2 of BRD3, exhibiting selectivity over BD1 of up to 23-fold [90]. However, previous studies of BRD3 that showed that its recruitment to acetylated sites on GATA1 is mediated by BD1 [91], suggesting the selective inhibition of RVX-208 may cause drugs nullity. Considering the important role ApoA1 played in hepatocellular carcinoma, and chemical inhibition of BDs has been associated with ApoA1 up-regulation, RVX-208 can be used as drugs of hepatocellular carcinoma. In addition, other potent BET inhibitor, JQ1 has strongly stimulated ApoA-I production in Hep-G2 cells in a post-translational regulation manner [92], making it appealing of developing multi-target inhibitors in hepatocellular carcinoma (Figure 4A).

#### BRDT Inhibitors

BRDT, as a tesis-specific BET family member, have been observed the selective function of its two BDs, in which deletion of BD1 in BRDT may result in abnormal spermatids and sterility [93]. Furthermore, altered histone modifications in mice have been observed in the BRDT promoter region of sub-fertile patients. A potent inhibitor of cyclin-dependent kinases (CDKs), Dinaciclib can interact with BRDT and bind in the ZA channel of BRD [94], and reporting the potential of Dinaciclib to act as protein-protein inhibitors of bromodomains, and considering Dinaciclib has advanced to Phase III clinical trials for the treatment of leukemia [91], the new findings may provide a new structural framework for the design of next-generation bromodomain inhibitors using the vast chemical space (Figure 4B).

## CONCLUSIONS

Bromodomain, as acetyl-lysine (Kac) “reader” domain, is part of the write−read−erase concept that has been linked with the transfer of epigenetic information. As part of the bromodomain-containing proteins, conserved BET family proteins processed dual-BD and an ET domain, suggesting a better structure feature for drug development. BET proteins mediate PPI network between diverse arrays of partners, and function as mitosis bookmark, protein scaffold and chromatin regulator in cellular processes. The information mentioned above has provided unique amino-acid BRDs signature and ability to regulating crucial cancer-regulation genes, revealing good potentials for drug targets in all subfamilies, thereby indicating that potent inhibitors targeting BET bromodomains would be developed.

Extensive studies have explored small-molecule inhibitors of BET family proteins for cancer therapy. Five small-molecule inhibitors have been used in clinical trials, such as I-BET762 in NMC and OTX-015 in hematologic cancers, inspiring researchers for further studies of BET inhibitors. Some hotspot inhibitors like JQ1 and I-BET151, I-BET 762 have been reported, which promote the clinical trial of these potential drugs, as well as gain more detailed information of the function and regulation activity of BET family. The experimental validation of pan-BET inhibitors have been further explored in different cancers, such as JQ1 and I-BET151, and experiments have validated that combination use of BET inhibitor and other drugs may reduce the drug resistance and raise the sensitivity towards other therapy. As a trend of current pharmaceutical design, developing multi-target inhibitors of BET family and combination use of BET inhibitors with other drugs would largely reduce the possibility of drugs resistance.

Hitherto, the limitation of BET proteins inhibitors also emerged, the negative finding of RVX-208 is disappointing for the potency and selectivity of this agent have not been disclosed in previous literatures. The selective inhibition of RVX-208 towards BD2 rather than BD1 in BRD3 suggests that specific targeting may be able to perturb and modulate BET function in a context-dependent manner. And notably, little study has been conducted about the diverse function of two BDs in one BET protein, except in BRDT that researchers confirmed that deletion of the first bromodomain in BRDT in mice is sufficient to confer sterility by blocking BRDT-dependent sperm maturation. Due to the alternative splicing, every BET family protein processes different isoforms, but the exact function and expression reason of these isoforms haven't been fully studied. Therefore, further development of domain and isoform specific inhibitors need to be necessary for unravelling the exact roles of BET bromodomains in gene transcription. And, the alternative expression levels of BET proteins in various cancers still need to be further discovered. However, BET proteins mediated PPI network have not been fully unraveled; therefore, development of mechanism towards PPI of BET may be a promising studying flied, which may also benefit the drug development to BET inhibitors.

In conclusion, rapid success has been achieved with the BET family of bromodomains, and numbers of potent small-molecule inhibitors have been reported. Inspiring findings that various inhibitors bear their own anti-tumor abilities have been enlightening us for further designing and discovering more new potential BET inhibitors. Moreover, clinical studies and mechanism elucidation of such inhibitors have made it clear that they would be invaluable tools for dissecting the biological roles of bromodomains and being potentially of great value as therapeutic leads.
